# Developing and validating machine learning models to predict vaccine hesitancy and literacy among adults in the United States

**DOI:** 10.3389/fpubh.2026.1669058

**Published:** 2026-03-11

**Authors:** Yi Zheng, Paula M. Frew, Dong Wang, Yan Song, Oscar Patterson-Lomba, Arshya Feizi, Tayler Li, Amanda L. Eiden

**Affiliations:** 1 Merck & Co., Inc., Rahway, NJ, United States; 2 Analysis Group, Inc., Boston, MA, United States

**Keywords:** health behavior, health literacy, machine learning, pediatric vaccination decision-making, surveys, vaccination, vaccination confidence, vaccine hesitancy

## Abstract

**Background:**

Vaccine hesitancy and literacy are multifaceted and context-specific phenomena that affect vaccination uptake. Comprehensively examining the simultaneous effects of various factors influencing vaccine hesitancy and literacy remains a challenge. This study aimed to better understand key determinants of adults’ vaccination decision-making, regarding both their own vaccinations and those of their children, using different machine learning algorithms to analyze survey data.

**Methods:**

A cross-sectional survey of US adults was conducted in 2022. Participants were categorized based on whether they had children under age 18 (“parents,” *N* = 692) or not (“adults,” *N* = 1,183). Survey responses were analyzed using multiple machine learning algorithms (logistic regression, decision tree, random forest, extreme gradient boost [XGBoost], support vector machine, and neural network) to predict adults’ and parents’ hesitancy toward vaccination, and literacy about their own or their children’s vaccination. Potential predictors included demographics, health literacy, information-seeking behavior, attitudes, and beliefs. Model performance was evaluated using F1 score and area under the receiver operating characteristic curve (AUROC) or the precision-recall curve (AUPRC). Feature importance was evaluated using Shapley values.

**Results:**

Among parents making vaccination decisions for their children, the random forest model achieved the highest predictive performance for vaccine hesitancy (F1 = 0.86, AUROC = 93.0%), and the XGBoost model performed best when predicting vaccine literacy (F1 = 0.64, AUPRC = 81.3%). Based on these models, the belief that “there is no need for my child to get vaccinated because everybody else does” emerged as the strongest predictor of hesitancy among parents, whereas low familiarity with the pediatric vaccination schedule was the main predictor of low literacy. Among adults making vaccination decisions for themselves, the XGBoost outperformed other models for both vaccine hesitancy (F1 = 0.77, AUROC = 90.3%) and vaccine literacy (F1 = 0.80, AUPRC = 86.0%). According to this model, having received an influenza vaccine was the strongest predictor of non-hesitancy among adults, and low familiarity with the adult vaccination schedule was the strongest predictor of low literacy.

**Conclusion:**

This study demonstrated the effectiveness of machine learning approaches in analyzing robust survey data. These models identified key determinants of vaccine hesitancy and literacy, offering valuable insights into the behavioral and informational factors influencing vaccination decisions among US adults.

## 1 Introduction

Vaccines are among the most cost-effective clinical preventive services, with a high economic and humanistic return on investment (1). However, in many parts of the world, individuals question the value, efficacy, and safety of vaccines and refuse vaccination for themselves or their children (2, 3).

Vaccine hesitancy, defined as a “delay in acceptance or refusal of vaccination despite availability of vaccination services,” is a multifaceted and context-specific challenge that varies by time, location, and vaccine type, affecting vaccination uptake (4). Uptake is also impacted by vaccine literacy, which is defined as the ability to “access, understand, and critically appraise and apply information about…vaccines,…in order to make informed decisions about vaccines…, and to appreciate the larger global impact of vaccines with respect to population health” (5). There is interplay between vaccine hesitancy and vaccine literacy through the demographic, attitudinal, behavioral, and experiential variables that affect them both (6–8).

Multiple tools are available to measure vaccine hesitancy and literacy, including the Vaccine Hesitancy Scale (9), the 3–7C models of vaccine literacy (5, 10), and the Parent Attitudes about Childhood Vaccines questionnaire (11). However, these tools consist of a limited number of items that do not capture the full array of factors that contribute to vaccine hesitancy and literacy. Examining the simultaneous role of the myriad variables that influence vaccine hesitancy and literacy is a complex task well suited to being handled by computational methods. Multi-factor survey data provide an ideal setting in which to develop machine learning algorithms that can identify key determinants of vaccine hesitancy and literacy. The aim of this study was to better understand factors that predict adults’ decision-making with respect to their own or their children’s vaccinations using conceptually different machine learning algorithms to analyze survey responses.

## 2 Materials and methods

### 2.1 Study design

The data source for this analysis was a cross-sectional, single visit, online survey of the United States (US) adult general population – a sample from National Health and Wellness Survey (NHWS) in 2022, which was previously described by Eiden et al. (12). The survey population consisted of US adults, categorized by whether they had children under age 18 (“parents,” *N* = 692) or not (“adults,” *N* = 1,183). Adults were defined as those that responded “no” to having children under the age of 18, or those that do not participate in the child’s vaccination decisions. Adults were queried regarding their own vaccination; parents were queried about vaccination of their children.

### 2.2 Outcomes

Specific survey questions were used to define hesitancy and literacy. Among parents, vaccine hesitancy was linked to the question, “Overall, how hesitant about childhood shots would you consider yourself to be?” Self-report of being “very hesitant,” “somewhat hesitant,” or “not sure” was defined as hesitancy, while answers of “not too hesitant” or “not at all hesitant” were defined as non-hesitancy. Among adults, vaccine hesitancy was linked to questions about the COVID-19 or flu vaccines, as a proxy for hesitancy toward annual or seasonal vaccines. For this subgroup, hesitancy was assessed with the question, “How would you describe your response when you were offered each of the following vaccines?” Answers of “hesitated but accepted” or “hesitated and refused” to either COVID-19 or flu vaccines were defined as hesitancy, and an answer of “accepted without hesitation” to both vaccines was defined as non-hesitancy. For both parents and adults, vaccine literacy was linked to the question, “How familiar are you with the vaccines your child/you should receive?” Answers of “moderately familiar” or “extremely familiar” were defined as high literacy, while answers of “somewhat familiar,” “slightly familiar,” or “not at all familiar” were defined as low literacy.

### 2.3 Features/predictors

Over 500 features, extracted from a structured set of survey questions and sub-questions, were evaluated as predictors of vaccine hesitancy or literacy. Topics covered included: experiences finding and assessing information regarding vaccination and their information-seeking and decision-making practices; interactions with healthcare providers about vaccines; beliefs about the efficacy, safety, and frequency of vaccines; level of trust in scientific and medical information from various sources; beliefs about the danger and severity of vaccine-preventable diseases; and beliefs about the benefits and convenience of vaccination. Demographics were also considered. Feature selection approaches were used to determine the most relevant features/predictors. Specifically, we used an information-based measure, maximal information coefficient (MIC), which captures both linear and non-linear relationships between the outcomes of interest and all the potential features (13). Models were trained using the top 10, top 25, top 40, top 50, top 80, and top 100 features in terms of MIC scores, calculated separately for each outcome. Comparing model performance across these progressively larger feature sets allowed us to assess potential overfitting: if increasing the number of features (that is, increasing model complexity) resulted in no improvement—or a decline—in predictive performance, this pattern was interpreted as evidence of overfitting. In addition, expert knowledge and survey design (e.g., removing questions that help define the outcome variables) were used to select features for the models.

### 2.4 Model development and validation

We employed 6 machine learning models: logistic regression (benchmark model), basic decision tree [classification and regression tree (CART)], random forest, extreme gradient boosting (XGBoost), support vector machine (SVM), and neural network (multilayer perceptron) (14, 15). To follow the best practice, the data were randomly split into a training set (80%) and a testing set (20%). The training set was used for hyperparameter tuning by a Bayesian optimization approach, where a range of values is selected for each hyperparameter, and the optimal values are extracted from within that range through an iterative process. A 10-fold cross validation was performed to select an optimal set of hyperparameters for each model (Table 1).

**Table 1:** Hyperparameters tuned for each model.

Algorithm	Hyperparameters
Logistic regression	Regularization (L1 or L2) C Tolerance Solver
Decision tree	Maximum depth of a tree Minimum samples to be a leaf Minimum samples to split internal node Function to measure the quality of a split The strategy used to choose the split at each node Minimum impurity decrease (Conceptually similar to gamma in XGBoost)
Random forest	Number of trees (i.e., estimators) Maximum depth of a tree Minimum samples to be a leaf Minimum samples to split internal node Function to measure the quality of a split Maximum features
Extreme gradient boost	Number of trees (i.e., estimators) Maximum depth of a tree Learning rate Minimum child weight Minimum loss reduction required to make a further partition on a leaf node of the tree (gamma) Subsample ratio of columns when constructing each tree Lambda (L2 regularization term on weights)
Support vector machine	Kernel C Gamma
Neural network	Solver Alpha Learning rate Activation function Maximum number of iterations Number of hidden layers Number of neurons per layer

Each hyperparameter was tuned using the Bayesian optimization approach.

The testing set was then used to perform the internal validation, and models with the best performance were chosen. To measure the goodness of fit and model accuracy, we calculated (i) area under the receiver operating characteristic curve (AUROC) which measures model’s overall discrimination ability, (ii) area under the precision-recall curve (AUPRC), a measure of the model’s intrinsic ability to distinguish between true and false positives in imbalanced datasets, (iii) accuracy (the proportion of the total predictions that are accurate), (iv) precision (the proportion of correctly predicted positive instances), (v) recall (the proportion of actual positive instances correctly identified by the model), and (vi) F1 score (the harmonic mean of precision and recall). All models were trained with the objective of maximizing the F1 score, which is commonly used to determine a model’s applicability to a real-world application and takes into account outcome imbalances in the data.

For the sake of robustness, 4 different training and testing sets generated by 4 random seeds were used to develop and validate the models. Performance metrics were averaged based on the 4 random training–testing sets; similarly, top predictors were determined based on average SHAP values. All models were trained using the top 10, top 25, top 40, top 50, top 80, and top 100 features in terms of MIC score, calculated separately for each outcome. For each outcome, we extracted important features from the model with the highest performance based on SHAP (SHapley Additive exPlanations) values, which explain the strength of each feature’s contribution to the overall prediction (16, 17). All analyses were conducted using Python 3.9 and R 4.2.

## 3 Results

### 3.1 Characteristics of the study population

The survey sample has been described in a previous publication (12). Briefly, 1,875 eligible adults were included in the analysis: 692 parents of children under age 18, and 1,183 adults without children aged <18 (Supplementary Table S1). The mean ± SD age of respondents was 46.7 ± 15.5 years, with older age in adults (50.9 vs. 39.5 in parents), and around half identified as female (53.8%, 1,008/1,875). Majorities of both parents and adults were White (66.3%, 459/692 and 74.4%, 880/1,183), college graduates (72.8%, 504/692 and 62.1%, 735/1,183), and employed (78.6%, 544/692 and 51.1%, 604/1,183), and adults generally had lower incomes than parents (Supplementary Table S1).

About half (49.7%, 344/692) of parents described themselves as vaccine hesitant (including those who ultimately accepted vaccination for their children), and 25.6% (177/692) were defined as having low vaccine literacy (Table 2). Among adults, 34.3% (406/1183) were vaccine hesitant, and 33.3% (394/1,183) had low vaccine literacy (Table 2).

**Table 2:** Distribution of vaccine hesitancy and literacy among parents and adults^A^.

Category	Parents [*n* = 692, n (%)]	Adults [*n* = 1,183, n (%)]
Vaccine hesitancy		
Yes	344 (49.7)	406 (34.3)
No	348 (50.3)	777 (65.7)
Vaccine literacy		
Low	177 (25.6)	394 (33.3)
High	515 (74.4)	789 (66.7)

^A^Values are presented as n (%).

### 3.2 Predictors of vaccine hesitancy and literacy among parents

**Figure 1:**
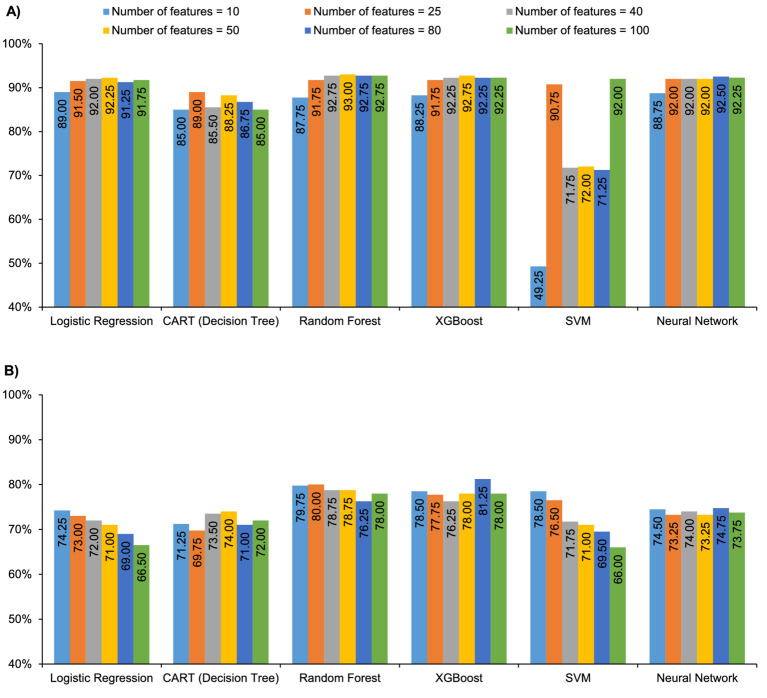
Model performance in parents: **(A)** AUROC for all models vs. number of features for vaccine hesitancy and **(B)** AUPRC for all models vs. number of features for vaccine literacy. AUPRC, area under the precision-recall curve; AUROC, area under the receiver operating characteristic curve; CART, classification and regression tree; SVM, support vector machine; XGBoost, extreme gradient boost.

Among parents making vaccination decisions for their children, the random forest model with 50 features performed the best in predicting vaccine hesitancy (F1 = 0.86, AUROC = 93.0%), and the XGBoost model with 80 features performed best when predicting vaccine literacy (F1 = 0.64, AUPRC = 81.3%) (Figure 1; Supplementary Table S2). The XGBoost model performed similarly well in predicting hesitancy using 40 or more features (Supplementary Table S2).

**Figure 2:**
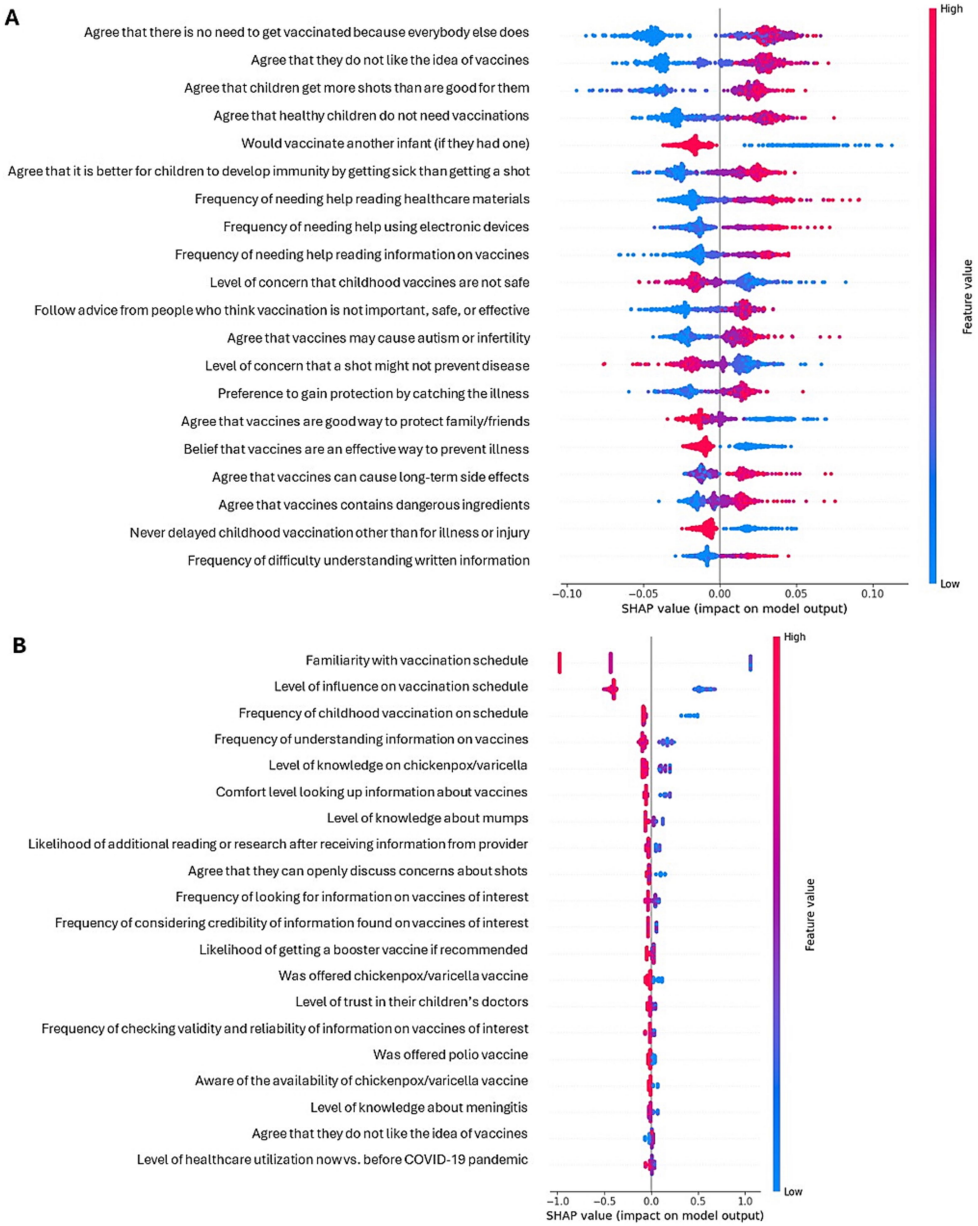
Shapley plots for predictors of vaccine hesitancy **(A)** and literacy **(B)** among parents.

Based on Shapley additive explanations (SHAP analysis) of these models, the belief that “there is no need for my child to get vaccinated because everybody else does” was the strongest predictor of vaccine hesitancy among parents, and low familiarity with the pediatric vaccination schedule was the strongest predictor of low literacy (Figure 2; Supplementary Table S3). Agreement with the statements that “I do not like the idea of vaccines for my child,” “children get more shots than are good for them,” and “healthy children do not need vaccinations” was also predictive of parental vaccine hesitancy, along with low agreement with wanting a new infant to get all the recommended shots. Low vaccine literacy among parents was also associated with a sense of little influence on their child’s vaccination schedule and with not knowing whether their child received their vaccines at the recommended times (or not allowing the child to be vaccinated on schedule), while high vaccine literacy was associated with understanding information obtained on vaccines and knowledge about chickenpox/varicella.

The top five predictors were generally consistent across different seeds. However, in the case of vaccine hesitancy, the belief that vaccines cause autism/infertility, concern for any one of the childhood shots not being safe, and agreeing that it is better for the child to develop immunity by getting sick rather than getting a shot also appeared in the top five predictors in some training/testing splits. In the case of vaccine literacy, the top five predictors in some splits included the ability to solve basic technical issues on their own, using vaccine/pharmaceutical companies’ web sites to learn about vaccines, comfort in looking up information about vaccines or learning about vaccines on their own, and reporting greater usage of healthcare services (by the child) than before the COVID-19 pandemic.

### 3.3 Predictors of vaccine hesitancy and literacy among adults

**Figure 3:**
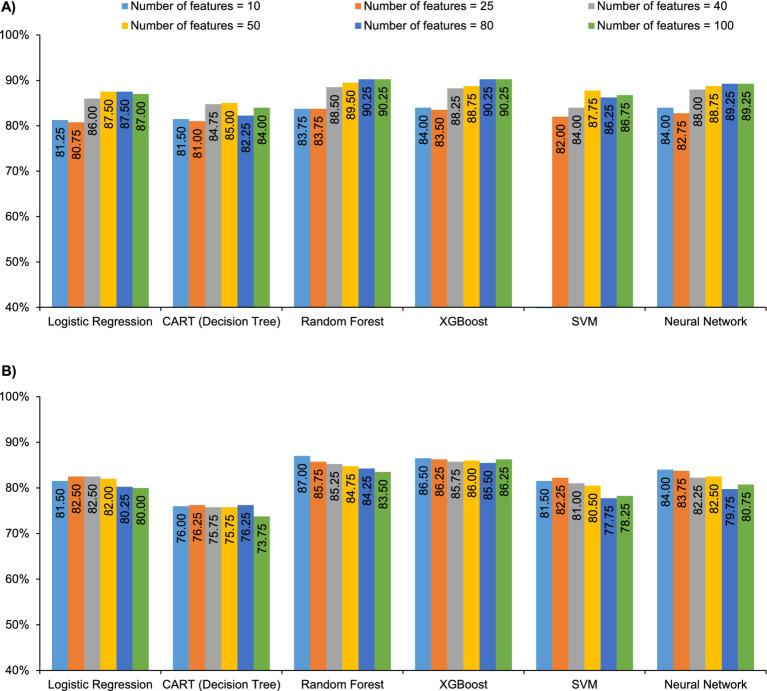
Model performance in adults: **(A)** AUROC for all models vs. number of features for vaccine hesitancy and **(B)** AUPRC for all models vs. number of features for vaccine literacy. AUPRC, area under the precision-recall curve; AUROC, area under the receiver operating characteristic curve; CART, classification and regression tree; SVM, support vector machine; XGBoost, extreme gradient boost.

Among adults making vaccination decisions for themselves versus parents for children, the XGBoost model with 100 features performed best when predicting vaccine hesitancy (F1 = 0.77, AUROC = 90.3%), and the XGBoost model with 50 features performed best when predicting low vaccine literacy (F1 = 0.80, AUPRC = 86.0%) (Figure 3; Supplementary Table S4). The random forest model with 80 features performed equally well when predicting hesitancy (Supplementary Table S4).

**Figure 4:**
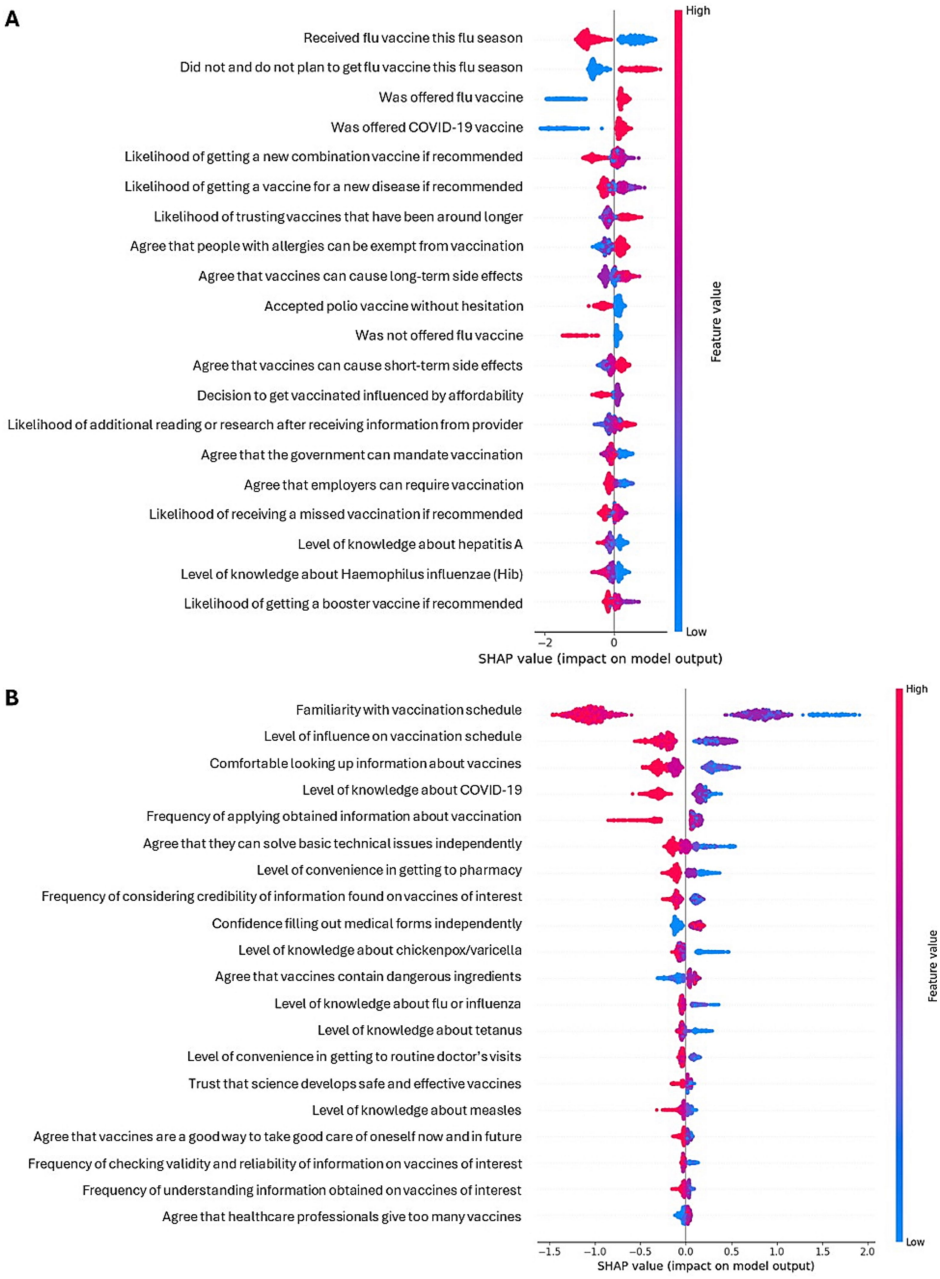
Shapley plots for predictors of vaccine hesitancy **(A)** and literacy **(B)** among adults.

Based on SHAP analysis of these models, the strongest predictor of non-hesitancy among adults was having received an influenza vaccine, and the strongest predictor of low literacy was low familiarity with the adult vaccination schedule (Figure 4; Supplementary Table S3). Other predictors of vaccine non-hesitancy were not being offered (or not recalling whether they were offered) an influenza or COVID-19 vaccine, and being more likely to accept a new vaccine that combines multiple vaccines into one, single vaccination. Conversely, answering “no and I do not plan to” when asked if they had received a seasonal flu vaccine during the current flu season was predictive of vaccine hesitancy. Other predictors of low vaccine literacy were a sense of little influence on the vaccination schedule and a lack of comfort when looking up information about vaccines or learning about vaccines, while high vaccine literacy was associated with awareness of the availability of a COVID-19 vaccine and high likelihood of applying information obtained on vaccines to make decisions regarding vaccination. In the case of vaccine literacy predictors, understanding the information obtained on vaccines and having the ability to solve basic technical issues also appeared among the top five predictors in some training/testing splits.

## 4 Discussion

This study used machine learning techniques to identify demographic, attitudinal, behavioral, and experiential factors that are associated with vaccine hesitancy and vaccine literacy among US adults, both for vaccinating themselves and their children. Among the 6 algorithms evaluated, the XGBoost model consistently demonstrated the highest predictive performance across outcomes, although other models, such as random forest, also had good prediction performance.

This study is novel in both the richness of its data and the breadth of its methodological approach. By analyzing over 500 features derived from survey responses, it is uniquely well-suited for generating evidence for understanding the factors that shape adults’ vaccination decision-making for themselves and their children. In addition, the study systematically applied and compared a wide range of machine learning algorithms, not only to identify the most predictive models but also to illustrate the value of advanced methods in uncovering important factors for vaccine hesitancy and literacy based on individual characteristics.

A few previous studies have used machine learning methods to analyze survey data regarding vaccination decisions. As an example, Lincoln et al. used a random forest algorithm to identify predictors of COVID-19 vaccine hesitancy derived from a multi-instrument survey (>100 items) of a cohort of 2,510 adults from 5 high-income nations (Australia, Germany, Hong Kong, the United States, and the United Kingdom) (18). They found that conspiracy beliefs about vaccines were the most influential predictors, followed by other specific and general measures of mistrust. Similarly, Bikaki et al. used machine learning techniques to analyze survey responses from 274 Texans regarding their intentions to receive a COVID-19 vaccine booster dose (19). Twenty-three survey questions on health and healthcare, trusted information sources, and preventive behaviors were analyzed by decision tree, logistic regression, and SVM methods after recursive feature elimination, and accuracy of the model was assessed by the Matthews correlation coefficient. This study found that confidence in the vaccine’s safety and trust in social media contacts were the strongest predictors of intention to receive a COVID-19 booster dose. Although direct comparisons of our results to these studies are limited due to differences in populations, survey instruments, and analytical methods, our study adds to the growing body of work demonstrating the utility of complex machine learning models (e.g., tree-based methods, SVMs, neural networks) in uncovering non-linear relationships that may be missed by simpler statistical approaches such as logistic regression.

A recently published manuscript described the use of a traditional logistic regression (i.e., inference-driven, as opposed to the prediction-driven approach used herein for the benchmark logistic model) to identify factors associated with vaccine hesitancy in the same parent population described in this study (*n* = 692) (20). In that study, factor analysis was used to reduce the number of survey questions to a clustered set of 8 factors. The factor most strongly associated with vaccine hesitancy was *negative beliefs about vaccines*, which included attitudinal variables such as “Healthy children do not need vaccinations,” “There is no need for my child to get vaccinated because everybody else does,” and “I do not like the idea of vaccines for my child.” The factor most strongly associated with low vaccine literacy was *trouble understanding information from healthcare providers (HCPs)*, which included experiential variables of finding text too hard to understand and needing a long time when reading vaccination information provided by an HCP. Our findings on vaccine hesitancy and literacy using machine learning were consistent with those from traditional logistic regression. “I do not like the idea of vaccines for my child” and “there is no need for my child to get vaccinated because everybody else does” appeared among the top features associated with parental vaccine hesitancy in this study, while difficulty understanding vaccine information was a key predictor of low parental vaccine literacy.

Predictors of vaccine hesitancy this study varied between adults making decisions for their children (the “parent” cohort) and those making vaccination decisions for themselves (the “adult” cohort), whereas predictors of vaccine literacy were largely consistent across these groups. The belief that “there is no need for my child to get vaccinated because everybody else does” was the strongest predictor of vaccine hesitancy among parents, while for adults the strongest predictor of non-hesitancy was having received a recent influenza vaccination. In contrast, for both parents and adults, the most salient predictor of low vaccine literacy was low familiarity with the vaccination schedule (either pediatric or adult). Whether these differences reflect real attitudinal distinctions between the adult and parental decision-making remains an open question, in part because the factor identified as the strongest predictor of vaccine literacy was conceptually very similar to the survey question used to define vaccine literacy in this study (i.e., both were linked to familiarity with the vaccine or vaccine schedule).

Incorporating multiple machine learning approaches into predictive modeling is crucial, especially when the underlying relationships between variables are complex or not well understood, as in the case of vaccine hesitancy, literacy, and the myriad potential predictors in our survey data. Different algorithms rely on distinct assumptions and mechanisms; for instance, logistic regression models linear relationships, while tree-based methods like random forests or XGBoost can capture intricate non-linear interactions. As a result, their abilities to handle challenges like high dimensionality, multicollinearity, class imbalance, and noisy features can vary widely. These differences can impact not only prediction accuracy but also the interpretation of predictor importance and the generalizability of results. Comparing the performance of multiple machine learning models helped us identify an approach that was appropriate for the data and research objectives, reducing the risk of relying on a pre-selected model that could be mis-specified or suboptimal. Our model comparison results suggested modest performance differences across algorithms, indicating that while advanced machine learning approaches offer improved predictive accuracy, especially for vaccine literacy, simpler models like logistic regression could still provide useful predictions for both vaccine hesitancy and literacy.

In this study, the term “predictors” refers to variables included as inputs in the machine learning models for predicting vaccine hesitancy and literacy. This terminology follows standard usage in predictive modeling and does not imply temporal or causal relationships and should not be interpreted as causal. Also, while this study demonstrates that ML models can serve as useful tools for identifying empirical predictors of vaccination attitudes and behaviors among US adults, ML cannot replace psychometric validation or theory-based approaches; however, it can complement them by efficiently revealing patterns and associations that may inform future, hypothesis-driven research.

The study has several limitations. The survey sample may not fully reflect the broader population. While participant quotas were established to mirror census distributions by region and race, the NHWS includes relatively limited representation from the most underserved populations, and the need for internet access likely excluded additional individuals. As a result, the findings may not be fully representative of all adults and parents in the US. A key limitation of the study design lies in the operational definitions of vaccine hesitancy and literacy, which may not have fully captured the complexity of these constructs. Unlike prior research that employed multi-item scales (5, 9–11, 21, 22), our study used single-item survey questions, resulting in comparatively simpler definitions. Furthermore, vaccine hesitancy among adults was assessed specifically in relation to seasonal flu and COVID-19 vaccines only, while hesitancy among parents was self-reported and thus subject to perceptual bias. These design choices may limit the comparability of our findings to those of other studies. However, consistent definitions within the study enabled valid comparison across the different machine learning algorithms, which was the main purpose of the analysis. A second limitation pertaining to the machine learning methods was that the division of training/testing data may influence which variables emerge as predictors. To address this, we used 4 different random seeds, and comparisons of Shapley values demonstrated that the top 5 predictors remained largely stable across runs, with the few exceptions noted above. Also, the performance metrics presented in the analysis did not include a measure of variability (e.g., 95% confidence intervals), and feature-group ablation studies were not conducted to quantify the relative importance of different domains of predictors. Finally, this analysis did not distinguish between vaccine hesitancy and literacy related to seasonal vs. routine vaccinations, which has been shown to exist in previous studies (20).

Future studies could build on this work by using survey instruments tailored to specific vaccines or vaccine-preventable diseases, thereby enabling more refined insights into the drivers of vaccine hesitancy and literacy. Also, validating the findings of this study in alternative datasets will be important to assess and strengthen its generalizability beyond this specific survey population. However, to our knowledge, the level of feature richness captured in this dataset is not currently available in other publicly available sources. Finally, developing a formal framework for evaluating and selecting prediction models according to pre-specified performance metrics would be a valuable direction for future research.

## 5 Conclusion

In conclusion, this study demonstrates that machine learning models can be effective tools for identifying predictors of vaccination attitudes and behaviors among US adults regarding decision-making for both personal and child vaccination. The factors identified align with intuitive reasoning and are conceptually consistent with previous studies that used traditional methods to identify predictors of hesitancy and literacy. Machine learning represents a promising approach for future analyses of complex, multi-item survey data related to vaccine hesitancy and literacy.

## Data Availability

The original contributions presented in the study are included in the article/Supplementary material, further inquiries can be directed to the corresponding author.
