# Supplementary material for: Developing and validating machine learning models to predict vaccine hesitancy and literacy among adults in the United States
Source: Front Public Health. 2026 Mar 11;14:1669058. doi: 10.3389/fpubh.2026.1669058 (PMC13013467; doi:10.3389/fpubh.2026.1669058)
Supplement: Supplementary file 1 [file Table_1.docx]

Supplementary Material

# Supplementary Tables

Supplementary Table S1. Demographic characteristics of survey respondents ^A^

|  | Total  (N=1875, n (%)) | Adults without children <18 years  (n=1183, n (%)) | Parents with children <18 years  (n=692, n (%)) | P-value ^B^ |
| --- | --- | --- | --- | --- |
| Sex assigned at birth |  |  |  |  |
| Female | 1025 (54.7) | 631 (53.3) | 394 (56.9) | 0.131 |
| Self-identified gender |  |  |  |  |
| Male | 847 (45.2) | 551 (46.6) | 296 (42.8) | 0.605 |
| Female | 1008 (53.8) | 622 (52.6) | 386 (55.8) |  |
| Prefer not to say | 4 (0.2) | 0 (0.0) | 4 (0.6) |  |
| Age, mean (SD) | 46.72 (15.47) | 50.92 (17.13) | 39.54 (8.06) | <0.001 |
| Community of residence |  |  |  |  |
| Large urban or city area (50,000 people or more) | 669 (35.7) | 330 (27.9) | 339 (49.0) | <0.001 |
| Suburban area (between 2,500-49,999 people) | 904 (48.2) | 640 (54.1) | 264 (38.2) |  |
| Rural area | 302 (16.1) | 213 (18.0) | 89 (12.9) |  |
| People within household including respondent, mean (SD) | 2.73 (1.44) | 2.05 (1.07) | 3.88 (1.26) | <0.001 |
| Region of residence |  |  |  |  |
| Northeast (New England and Mid-Atlantic regions) | 367 (19.6) | 233 (19.7) | 134 (19.4) | 0.812 |
| Midwest (East North Central and West North Central regions) | 450 (24.0) | 299 (25.3) | 161 (23.3) |  |
| South (South Atlantic, East South Central and West South Central regions) | 671 (35.8) | 416 (35.2) | 255 (36.8) |  |
| West (Mountain and Pacific regions) | 377 (20.1) | 235 (19.9) | 142 (20.5) |  |
| Race |  |  |  |  |
| White | 1339 (71.4) | 880 (74.4) | 459 (66.3) | <0.001 |
| Black | 209 (11.1) | 140 (11.8) | 69 (10.0) |  |
| Asian | 113 (6.0) | 63 (5.3) | 50 (7.2) |  |
| Hispanic | 53 (2.8) | 31 (2.6) | 92 (13.3) |  |
| Other (Native Hawaiian/Pacific Islander and American Indian/Alaska Native) | 161 (8.6) | 69 (5.8) | 92 (13.3) |  |
| Yearly household income |  |  |  |  |
| ≤$49,999 | 674 (35.9) | 527 (44.5) | 147 (21.2) | <0.001 |
| $50,000 to $99,999 | 615 (32.8) | 371 (31.4) | 244 (35.3) |  |
| ≥$100,000 | 534 (28.5) | 243 (20.5) | 291 (42.1) |  |
| Decline to answer | 52 (2.8) | 42 (3.6) | 10 (1.4) |  |
| Highest level of formal education completed |  |  |  |  |
| Did not attend college or attended but no degree | 634 (33.8) | 446 (37.8) | 188 (27.2) | <0.001 |
| College graduate | 1239 (66.1) | 735 (62.1) | 504 (72.8) |  |
| Decline to answer | 2 (0.1) | 2 (0.2) | 0 (0.0) |  |
| Employment status |  |  |  |  |
| Employed (full-time/part-time/self-employed) | 1148 (61.2) | 604 (51.1) | 544 (78.6) | <0.001 |
| Unemployed | 638 (34.0) | 508 (42.9) | 130 (18.9) |  |
| Disability | 54 (2.9) | 47 (4.0) | 7 (1.0) |  |
| Decline to answer | 35 (1.9) | 24 (2.0) | 11 (1.6) |  |
| Marital/Cohabiting status |  |  |  |  |
| Married/Living with partner | 1111 (59.2) | 534 (45.1) | 577 (83.3) | <0.001 |
| Single (ie, never married, divorced, separated, widowed) | 763 (40.6) | 649 (54.9) | 114 (16.5) |  |
| Decline to answer | 1 (0.1) | 0 (0.0) | 1 (0.1) |  |
| Currently have health insurance | 1,697 (90.5) | 1,070 (90.4) | 627 (90.6) | 0.910 |
| Type of primary insurance |  |  |  |  |
| Commercial | 1097 (64.6) | 607 (56.7) | 490 (78.1) | <0.001 |
| Medicaid | 167 (9.8) | 107 (10.0) | 60 (9.6) |  |
| Medicare | 372 (21.9) | 318 (29.7) | 54 (8.6) |  |
| Military (VA, CHAMPUS, TRICARE) | 27 (1.6) | 20 (1.9) | 7 (1.1) |  |
| Not Sure | 34 (2.0) | 18 (1.7) | 16 (2.6) |  |
| No insurance | 178 (9.5) | 113 (9.6) | 65 (9.4) |  |

SD, standard deviation

^A^ Values presented as n (%) unless indicated otherwise.

^B^ P values for the bivariate analysis of adults without children <18 years vs. parents with children <18 years.

Supplementary Table S2. Performance metrics for the models predicting vaccine hesitancy and literacy in parents (attached as Excel file)

Supplementary Table S3. Shapley values for the top predictors of vaccine hesitancy and literacy (attached as Excel file)

The Excel file contains the mean absolute Shapley values (across 4 seeds) for each feature for the top performing model for each of the 4 outcomes (vaccine hesitancy and literacy in the adult and parent population).

- Columns A-B are the question numbers and actual questions, respectively. Column C is populated if the question could have multiple answers, but that specific answer is what is informative (eg, answering “Not offered this vaccine” when asked “B102r2: COVID-19 - To your knowledge, have you [has your child] ever been offered a vaccine for any of the following (regardless of whether you received the vaccine or not)?”). Columns D-E and F-G are the Shapley values for the adult and parent populations, respectively. Note that if the Shapley value appears empty, it is because that feature was not included in the features identified by the model. (Note that the models have 100 or 50 or 80 features.)
- The highlighted cells indicate that this item appeared among the top 20 features. Each column (ie, outcome of interest) may be sorted from largest to smallest to view the top features relevant to predicting that outcome.
- The green font in some cells indicates that the question’s importance in predicting the outcome may be considered unexpected.

Supplementary Table S4. Performance metrics for the models predicting vaccine hesitancy and literacy in adults (attached as Excel file)
